# Silent Giant: A Case of Dedifferentiated Retroperitoneal Liposarcoma Presenting as an Incidental Mesenteric Mass

**DOI:** 10.7759/cureus.72549

**Published:** 2024-10-28

**Authors:** Danielle A Rowe, William B Bowers, Heather L Mateja, Alyson Morgan, David Thomas

**Affiliations:** 1 General Surgery, American University of Antigua, Osbourn, ATG; 2 General Surgery, Western Reserve Health Education/Northeast Ohio Medical University (NEOMED), Warren, USA; 3 General Surgery, Sharon Regional Medical Center, Sharon, USA

**Keywords:** case report, dedifferentiated liposarcoma, en bloc resection, exploratory laparotomy, giant liposarcoma, mesenteric mass, tumor surgery

## Abstract

Dedifferentiated liposarcoma (DDL) is a rare and aggressive subtype of liposarcoma, arising most commonly in the retroperitoneum but rarely presenting in the mesentery. Due to its rarity, mesenteric DDL poses significant diagnostic and therapeutic challenges, with few cases documented in the literature. Here, we report the case of a 76-year-old female admitted with acute respiratory failure secondary to pneumonia, during which an incidental finding of a large mesenteric mass was noted on computed tomography (CT) imaging. An elective exploratory laparotomy was performed one month later, revealing a large mesenteric tumor adherent to the terminal ileum, cecum, and ascending colon. En bloc resection, including right hemicolectomy and partial small bowel resection, was successfully performed. Pathology confirmed an intermediate-grade DDL. This case underscores the importance of considering mesenteric DDL in the differential diagnosis of large, asymptomatic intra-abdominal masses. Complete surgical resection remains the mainstay of treatment, with a critical focus on achieving negative margins to reduce recurrence risk. Given the aggressive nature of DDL, vigilant long-term follow-up is essential. This report contributes to the limited body of literature on mesenteric DDL and highlights the challenges in its diagnosis and management.

## Introduction

Liposarcoma (LPS) is a rare tumor arising from the mesenchymal cells that involve deep soft tissue [1,2]. The 2020 World Health Organization Classification of Tumors of Soft Tissue and Bone recognizes five major LPS subtypes: well-differentiated LPS (WDL)/atypical lipomatous tumor (ALT), dedifferentiated LPS (DDL), myxoid LPS, pleomorphic LPS, and myxoid pleomorphic LPS [3]. DDL occurs as a result of dedifferentiation of well-differentiated LPS [2,4,5]. DDL is extremely rare and occurs in less than 0.1 per 100,000 each year [6]. DDL typically occurs in adult males in the fifth to sixth decades of life [1,5] and is associated with more aggressive clinical behavior, propensity for local recurrence, the ability for metastasis, and a poor prognosis [4,5]. DDL most commonly occurs in the retroperitoneum, extremities, and trunk [4,7,8]. Mesenteric DDL involving the colon and small intestine is a rare occurrence, with infrequent reports in the literature that pose significant diagnostic difficulties [7,8].

Due to the rarity of this presentation, we present the case of a 76-year-old female who had an incidental finding of a mesenteric mass on computed tomography (CT) during hospitalization for acute respiratory failure. Exploratory laparotomy one month later revealed a 15 cm DDL, which was resected successfully. We present this article to raise awareness of this rare but life-threatening LPS and to highlight the significance of surgical intervention.

## Case presentation

**Figure 1:**
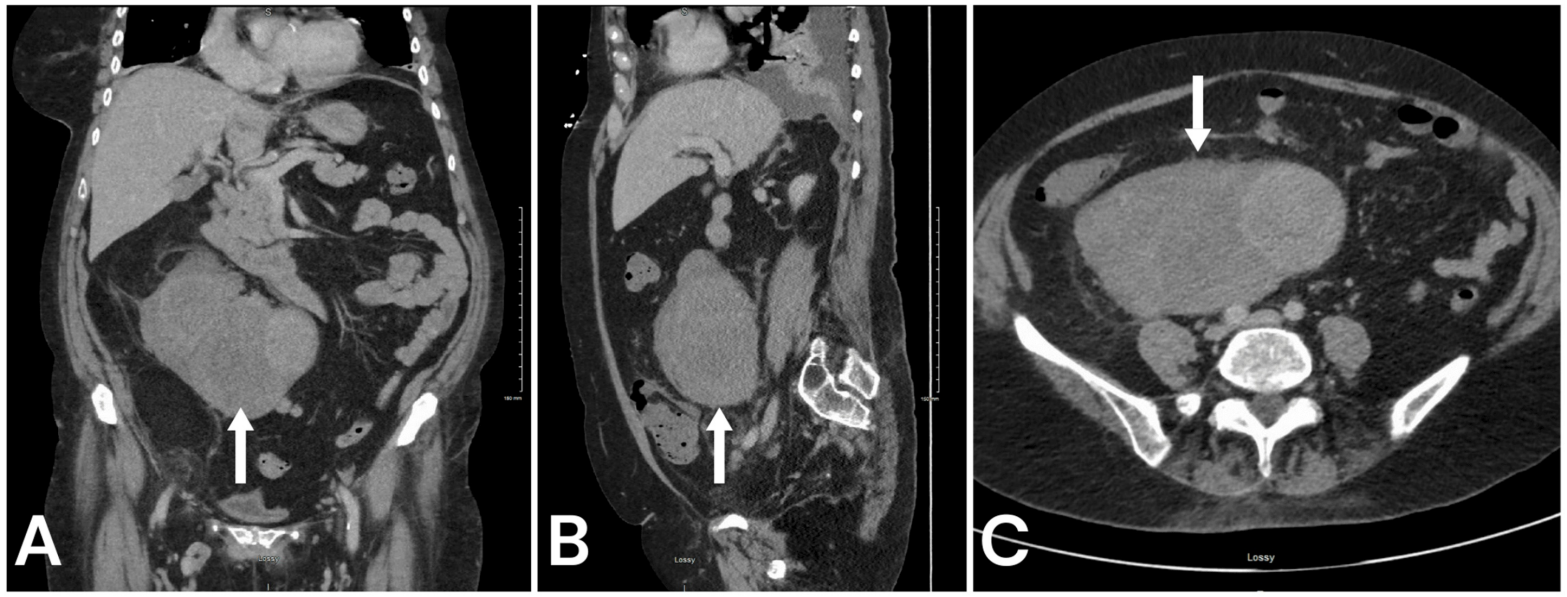
CT imaging with contrast demonstrating heterogeneous enhancement of the large mass (white arrows) located within the retroperitoneum and attached to the mesentery of the right colon. This appeared contiguous with the abdominal vein. No adenopathy or other enhancing lesions noted and no liver metastases. (A) Coronal. (B) Sagittal. (C) Axial.

A 76-year-old female, with a past medical history significant for atrial fibrillation on rivaroxaban, hypertension, hyperlipidemia, hypothyroidism, valvular heart disease, and glaucoma, was admitted to the hospital for acute respiratory failure secondary to pneumonia. During this admission, she reported abdominal pain and diarrhea, leading to a non-contrast and contrast-enhanced CT scan of the abdomen and pelvis (Figure 1). It revealed a large mesenteric mass measuring 15 x 9 x 15 cm, with heterogeneous density and a Hounsfield unit (HU) of 33. The mass was below the anterior aspect of the right kidney and extended caudally into the pelvis. Additionally, a fatty mass adjacent to or contiguous with the primary mass was identified in the right pelvis, measuring 5.7 x 6.5 cm.

Tumor markers, including cancer antigen (CA)-125, were elevated, suggesting a possible gynecological origin; however, the patient’s history of bilateral oophorectomy complicated the diagnostic interpretation. Despite the size of the mass, the patient denied significant prior symptoms such as abdominal pain, nausea, vomiting, chest pain, shortness of breath, or systemic symptoms such as fever, chills, or weight loss. She reported chronic liquid bowel movements, but no recent changes in her bowel habits.

The patient was referred to general surgery for further evaluation and management. Given the size, location, and imaging characteristics of the mass, a decision was made to proceed with surgical intervention. She was discharged home and followed up one month later for elective exploratory laparotomy. During the procedure, a large, firm, irregularly contoured pelvic mass was identified, adherent to the terminal ileum, cecum, and ascending colon. An en bloc resection of the mass was performed, including a right hemicolectomy and partial small bowel resection. The mass was closely associated with the right ureter, requiring careful dissection to avoid ureteral injury. Additional procedures included a total abdominal hysterectomy, omentectomy, and lymph node dissection. The operation was completed without intraoperative complications, and the patient was transferred to recovery in stable condition.

A gross examination of the resected specimen revealed a 15 cm DDL with satellite nodules arising in the mesentery and showing focal adhesions to the colonic serosa. The tumor was classified as intermediate grade (French Federation of Cancer Centers Sarcoma Group (FNCLCC) grade 2 of 3). Sixteen mesenteric lymph nodes were sampled, all of which were negative for metastatic involvement. The uterus and cervix were unremarkable, with only benign changes noted. Based on the surgical and pathological findings, the patient did not need to proceed with chemotherapy or radiation and has remained cancer-free as of a six-month follow-up, being followed closely by the oncology team.

## Discussion

DDL can be classified into two types: primary and secondary. Primary DDL is discovered at the time of the initial diagnosis, often arising from soft tissue unexpectedly [8]. It accounts for approximately 57% of liposarcomas found in the retroperitoneum [9]. Secondary DDL develops from a previously diagnosed well-differentiated liposarcoma that loses its specific tissue features during recurrence [8]. The ratio of primary to secondary DDL is roughly 9:1 [5]. Our patient represents a primary DDL, as it was determined upon the initial diagnosis.

The exact cause of DDL is unknown, and the exact gene mutation involved in its pathogenesis is still under investigation [1]. Common genetic abnormalities involved are the amplification of CDK4 and MDM2 cell cycle oncogenes [6,10]. Risk factors for developing liposarcoma include prior radiation therapy, damage to the lymphatic system, and exposure to toxic chemicals [1]. Our patient had no known exposure to these risks. Importantly, DDL can still occur in the absence of these factors, as highlighted by our case, indicating that the development of this tumor may be multifactorial and unpredictable [1].

The clinical presentation of DDL depends on the size and location of the tumor [11,12]. These symptoms usually arise from the displacement of nerves and vessels or compression of surrounding organs [8]. As the tumors enlarge, they can cause intestinal or urinary obstructions [8]. Many DDLs are asymptomatic until they reach significant sizes, with about 20% exceeding 10 cm at diagnosis [8]. In our patient, the 15 cm mass was an incidental finding during imaging for abdominal pain while hospitalized for an unrelated condition, demonstrating the tumor's ability to grow silently. She reported only minor symptoms of chronic diarrhea and recent abdominal pain, which did not correlate with the tumor's size and extent. This aligns with the literature, which notes that symptoms such as diffuse abdominal pain, weight loss, or mass effects from compression of nearby organs tend to occur in advanced cases [6,8,12]. Patients may also report a freely mobile mass, abdominal distension, and, in rare cases, intussusception or symptoms mimicking conditions such as diverticulitis [13].

Magnetic resonance imaging (MRI) remains the diagnostic tool of choice for soft tissue lesions, as it can demonstrate the fatty nature of the tumor [6]. To date, there are no unifying diagnostic criteria to differentiate between the different types of liposarcomas [10]. There are however features that may be present in diagnostic imaging suggesting that, one type over the other, definitive diagnosis includes open biopsy with subsequent histologic and immunohistochemical analysis [1,2]. MRI characteristics of DDL show two components: a well-differentiated liposarcoma (WDL) with high signal intensity on T1- and T2-weighted images, and a larger dedifferentiated component with prolonged T1 and T2 times [6]. This dedifferentiated part may show hemorrhage or necrosis, and gadolinium contrast enhancement varies [6]. CT imaging is important to assess the size and invasion of local structures [12]. In some instances, as in our case, the mass is discovered incidentally during imaging performed for unrelated clinical concerns [2].

Complete surgical excision is the only curative option for DDL, regardless of the tumor’s size [1,6,8]. Achieving negative margins (R0 or R1 resection) is critical to improving overall survival and reducing recurrence rates, although this is more challenging in retroperitoneal and mesenteric DDL due to the proximity of vital neurovascular structures [6,8]. In our patient, en bloc resection of the mass, along with adjacent bowel and omentum, was performed successfully. The procedure also included careful dissection near the right ureter to avoid injury. This highlights the complexity of surgical management in cases where the tumor is closely associated with critical structures. While R0 margins are more feasible in extremity DDLs, mesenteric tumors present unique challenges due to their anatomical location [8].

Adjuvant chemotherapy and radiation therapy may be used to lower local recurrence rates in DDL [1,6,11], though they are often avoided in mesenteric cases due to the risk of complications such as enteritis [8]. Similarly, our patient did not receive additional therapy and has not had any recurrence as of her six-month follow-up. Given the aggressive nature of DDL and its propensity for local recurrence, postoperative surveillance is essential. Regular CT scans every three to four months for the first two years are recommended to monitor for recurrence [1,13].

## Conclusions

This case of mesenteric DDL highlights the rarity and diagnostic challenges of this aggressive soft tissue tumor. Despite its considerable size, the patient exhibited only minimal symptoms, which underscores the often-asymptomatic nature of DDL until it reaches a significant size. Early recognition and prompt surgical intervention are critical, as complete surgical resection remains the cornerstone of curative treatment. The successful en bloc resection performed in this case, including multivisceral excision, allowed for optimal oncologic outcomes. Given the poor prognosis associated with DDL and its propensity for local recurrence, close follow-up is essential to monitor for recurrence.
